# Role of Repressive Histone Lysine Demethylases and Methylases in Susceptibility to Depression Using a Novel Progressive Social Defeat Stress Mouse Model

**DOI:** 10.1007/s10571-025-01597-3

**Published:** 2025-08-11

**Authors:** Arpan Mukhoti, P. K. Annapoorna, Ashutosh Kumar, Pratishtha Wadnerkar, Ayesha Atqa Khan, Salil Saurav Pathak, Sumana Chakravarty, Arvind Kumar

**Affiliations:** 1 CSIR-Centre for Cellular and Molecular Biology, Hyderabad, India; 2 Applied Biology Division, CSIR-Indian Institute of Chemical Technology, Hyderabad, India; 3 Academy of Scientific and Innovative Research, Ghaziabad, UP 201002 India; 4 Epigenetics & Neuropsychiatric Disorders Laboratory, CSIR-Centre for Cellular and Molecular Biology, Uppal Road, Habsiguda, Hyderabad, Telangana State 500007 India

**Keywords:** Resilience, Phf8, H3K9me2/H3K27me2, Dentate gyrus, Hippocampus, Nucleus accumbens, Progressive social defeat stress (PSDS)

## Abstract

Major depressive disorder (MDD) results from repeated and constant exposure to stress over prolonged periods. The highly variable response to stress and the low heritability suggests that MDD has a strong epigenetic basis. Studies show global dysregulation of histone modifications in both susceptible and resilient animals after chronic stress suggesting involvement of epigenetics in stress response in the brain. Given that the hippocampus and dentate gyrus (DG) show epigenetic changes in neurogenesis in Rodent models of stress that is known to be highly affected in MDD, we hypothesized that epigenetic changes might be involved in the advent of depressive phenotype during the progressive stress paradigm. To study the stress progression into the depression-like phenotype at the molecular level, we designed a novel progressive social defeat stress (PSDS) paradigm based on the popular chronic social defeat stress (CSDS) paradigm but involving only 5 days of defeat stress. Our molecular studies revealed consistent downregulation of H3K9me2 marks in the hippocampus and DG after the 4th day of stress while H3K27me2 showed an early upregulation in the hippocampus and a late downregulation after the 5th day of stress in the DG. In parallel, an early increase in *phf8* and *phf2* in hippocampus and DG, respectively, was observed. These findings of variable changes like repressive histone methylation marks and expression of corresponding demethylase genes after different durations of defeat stress, led to better understanding of the important role epigenetics play in stress progression into depression at molecular level in establishing resilient and susceptible phenotypes.

**Supplementary Information:**

The online version contains supplementary material available at 10.1007/s10571-025-01597-3.

## Introduction

Major depressive disorder (MDD) is a debilitating psychiatric disorder which affects 35% of the global population (Cai et al. 2023). Multiple studies show brain structure and chemistry alterations in MDD (Kong et al. 2023; Xie et al. 2023). For a long period of time changes in neurotransmitter levels were thought to be responsible for depressive disorder and most treatment methods were directed to alter these changes. But recent studies have shown a much deeper molecular basis of depressive disorder involving both genetic and epigenetic factors (Nestler et al. 2002). Meta-analyses have shown only 31–42% heritability in depressive disorders (Sullivan et al. 2000). Even monozygotic twins show about 50% discordance in the occurrence of MDD suggesting that the susceptibility is not entirely genetic (Fraga et al. 2005).

Environmental factors such as exposure to stressful events, increase the risk of MDD (Hammen 2005; Kessler 1997). Stress response, however, shows a remarkable degree of individual variability with regards to vulnerability. Majority of the population exposed to environmental stress does not develop MDD (Dudley et al. 2011). These findings have led to the theory that MDD occurs as a result of interaction between genetic and environmental factors like stress (Kendler 1998; Vialou et al. 2013).

Furthermore, alterations in chromatin structure of the brain has been observed in rodents after exposure to chronic stress (Vialou et al. 2013) with global changes in levels of histone acetylation and Methylation (Covington et al. 2009; 2011; Weaver et al. 2004; Wilkinson et al. 2009). Histone acetylation and repressive methylation have been shown to play a partial role in stress resilience and susceptibility in mice, such as di-methylation of histone 3, lysine 9 (H3K9me2) in the nucleus accumbens (NAc) (Covington et al. 2011; Wilkinson et al. 2009). Although these epigenetic modifications show variation in their global and region specific prevalence; for example histone acetylation levels increase globally in depression, but HDAC inhibition specifically in NAc promotes resilience and induces effects similar to anti-depressants (Covington et al. 2009). Also H3K9me2 levels are high globally in depressive disorders but local inhibition or knockout of histone methyltransferases in NAc increases susceptibility to depression and activation of the same promotes resilience (Covington et al. 2011).

Hippocampus also plays an important role in MDDs, and reduced hippocampal volume is observed in patients of MDDs (Videbech and Ravnkilde 2004; Campbell et al. 2004), the magnitude of which correlates to the frequency of depressive episodes and length of time for which depression has remained untreated (MacQueen et al. 2003). Such alteration in the physiology of the hippocampus may lead to changes in other brain regions, that receive signals from the hippocampus, such as the prefrontal cortex (PFC), NAc (O’Donnell and Grace 1995; Maren and Hobin 2007; Seidenbecher et al. 2003; Lisman and Grace 2005), amygdala; which are associated with emotionality. It has been observed that most treatments or environmental interventions that; results in antidepressant like effect, promotes neurogenesis (Malberg et al. 2000; Madsen et al. 2000). This suggests that the dysregulation of the same might play an important role in depressive disorder, along with the dentate gyrus, which is present within the hippocampus and is the seat for adult neurogenesis in the brain.

A single event of restrain stress has been shown to be sufficient to induce changes in the methylation patterns of the hippocampus (Hunter et al. 2012). Also a single event of social defeat stress has been found to cause changes in dopaminergic neurotransmission in the NAc after 24 h of the stress (Nemets et al. 2022). Sub-chronic stress over 10 days has been shown to be sufficient to elicit changes in the miRNA transcriptome and behavioral changes associated with depression-like phenotype (Yoshida et al. 2022). Social defeat stress over varying duration shows that 10 days and 30 days of stress results in highly different transcriptome in brain (Reshetnikov et al. 2022). This suggests that; expression profile of the brain is highly variable based on the duration and the number of stress events. Also histone modifications such as methylation and serotonylation show differential levels not only in CSDS of variable duration but also in chronic depression phenotype (Reshetnikov et al. 2022; Torres-Berrío et al. 2024; Al-Kachak et al. 2024), suggesting an important role of histone modification in stress progression and advent of depression-like-phenotype.

These previous findings suggesting a differential effect of stress in the brain based on the number of stress events and their durations interested us in understanding stress progression into depression-like phenotype at a molecular level. To this end, in this paper we designed a variation of the social defeat stress protocol where variable number of stress is induced, till the advent of depression-like phenotype in C57 mice, and try to understand the molecular patterns of histone methylations to gain a better understanding of the establishment of the molecular patterns observed after chronic social defeat stress.

## Results

### Progressive Social Defeat Stress Induces Depression-Like Phenotype After Four Episodes of Stress

**Fig. 1:**
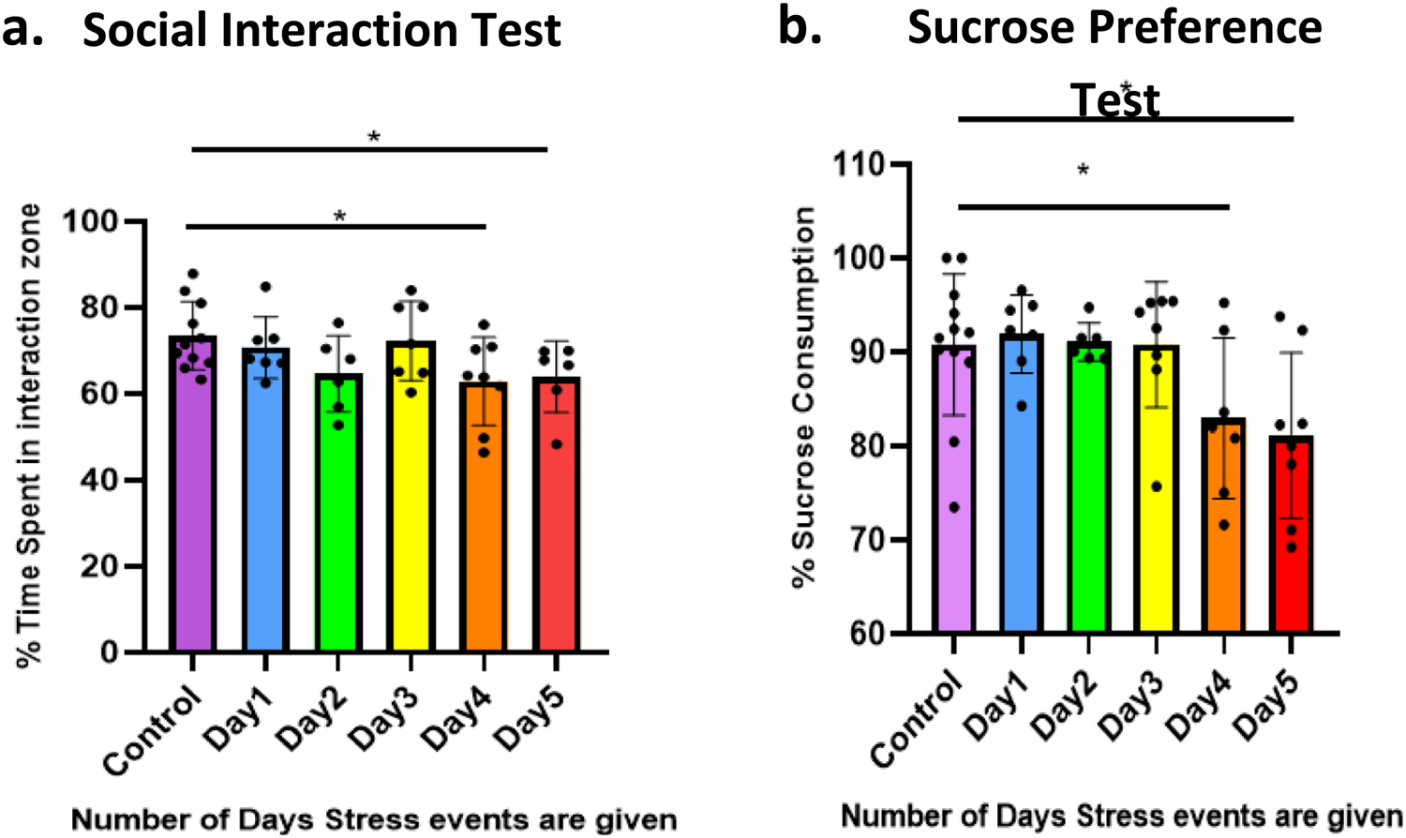
Results of behavior tests to assess depression-like phenotype in the PSDS model. **a** Social interaction (SI) test—graph shows percentage of SI which is the amount of time spent in the interaction zone with an unfamiliar CD1 mouse inside the box divided by amount of time spent in the zone with and without a CD1 inside the box; **b** sucrose preference test—graph shows percentage of sucrose preference which was calculated as a percentage of the ratio of amount of sucrose solution consumed by total fluid consumed during the last three days of defeat; Significance calculated using Student’s *t*-test; *p ≤ 0.05; data presented as mean ± SD (*n* = 8–9)

We designed the progressive social defeat stress (PSDS) protocol based on the chronic social defeat stress (CSDS) paradigm, with the goal of understanding the gradual molecular changes in the progression of stress-induced depression-like phenotype. The classic CSDS paradigm lasts 10 days with the mice receiving 5–10 min of defeat stress each day (Golden et al. 2011). Although, our lab has previously reported that a certain percentage of mice develop depression-like phenotype after 5 events of defeat stress (Pathak et al. 2017). We further sought to understand after how many exact defeat stress events do the mice start showing indication of a depression-like phenotype. For this purpose, the mice underwent behavior tests—sucrose preference test and social interaction test, after each stress event. Both these tests assess the reward perception of mice, correlated with anhedonia, the hallmark of depression. We observed that mice showed a significantly reduced sucrose preference and social interaction after they had undergone four defeat stress events. These Findings indicate an attenuated reward perception, characteristic of depression-like phenotype (Fig. 1a, b). Thus our results suggest that it takes at least four social defeat stress episodes in mice to induce depression-like phenotype.

### The Potential Role of Hippocampal Neurogenesis in the Progressive Social Defeat Stress Induced Establishment of Depression-Like Phenotype

**Fig. 2:**
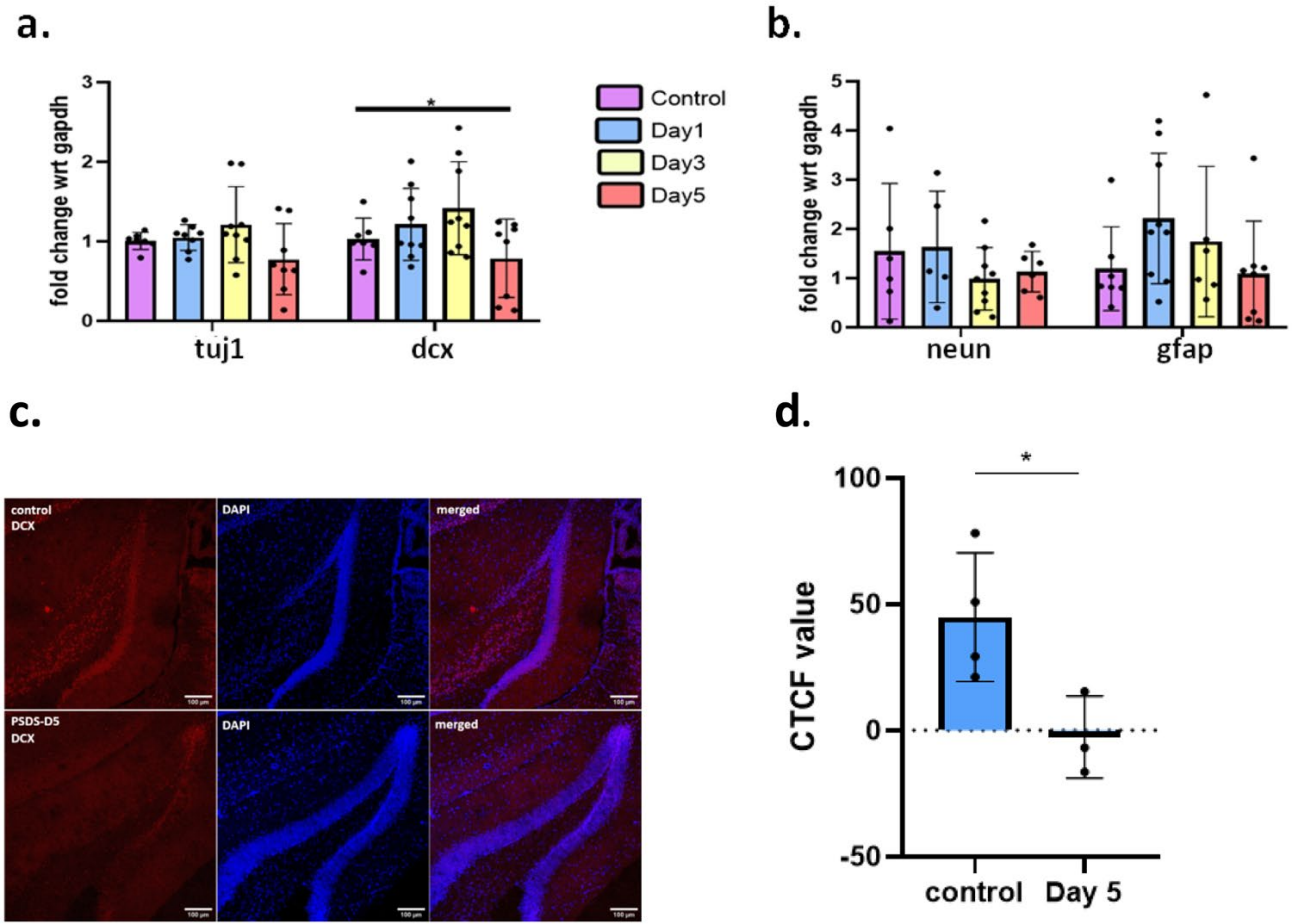
Expression levels of **a** early neuronal differentiation markers *tuj1, dcx;* **b** mature cell type markers *neun, gfap*; **c** representative images of DCX protein expression in dentate gyrus of mice from control group (upper panel) and PSDS Day 5 group (lower panel), cell nuclei were marked by DAPI; and **d** graphical representation of DCX expression as observed from the fluorescence intensity. For graphs representing relative fold change in mRNA of the genes; *gapdh* was considered as the housekeeping control; significance level was calculated using Student’s *t*-test; *p ≤ 0.05; data presented as mean ± SD (*n* = 8–9 for qPCR), (*n* = 3–4 for IHC)

There are several studies that have found a dysregulation in hippocampal neurogenesis in stress and depression (Van Bokhoven et al. 2011; Yun et al. 2010; Lucassen et al. 2010). To investigate the status of hippocampal neurogenesis in the PSDS induced depression model, we checked the mRNA expression levels of neurogenesis and neural and glial differentiation markers in the hippocampal neurogenic region DG after each stress event. It was observed that as the stress progressed, there was a significant downregulation of early neuronal differentiation marker, *dcx* (doublecortin) in the DG after the 5th stress event (Fig. 2a). The downregulation of DCX was confirmed by immuno-histochemistry (IHC) where we observed a decreased signal intensity in the DG of stressed mice when probed for DCX (Fig. 2c, d); *DCX* being considered a marker of early neurogenesis. Our finding suggests a reduction in hippocampal neurogenesis after five episodes of defeat stress. A non-significant reduction in the mRNA levels of another early mature neuron marker, *tuj1* (or beta 3 tubulin) was also found, while no significant changes were noted in mature neuron and glial markers, *neuN* and *gfap* (Fig. 2a, b).

### Dysregulation in the Global Levels of Repressive Histone Methylation in Hippocampus in the Progressive Social Defeat Stress Model

**Fig. 3:**
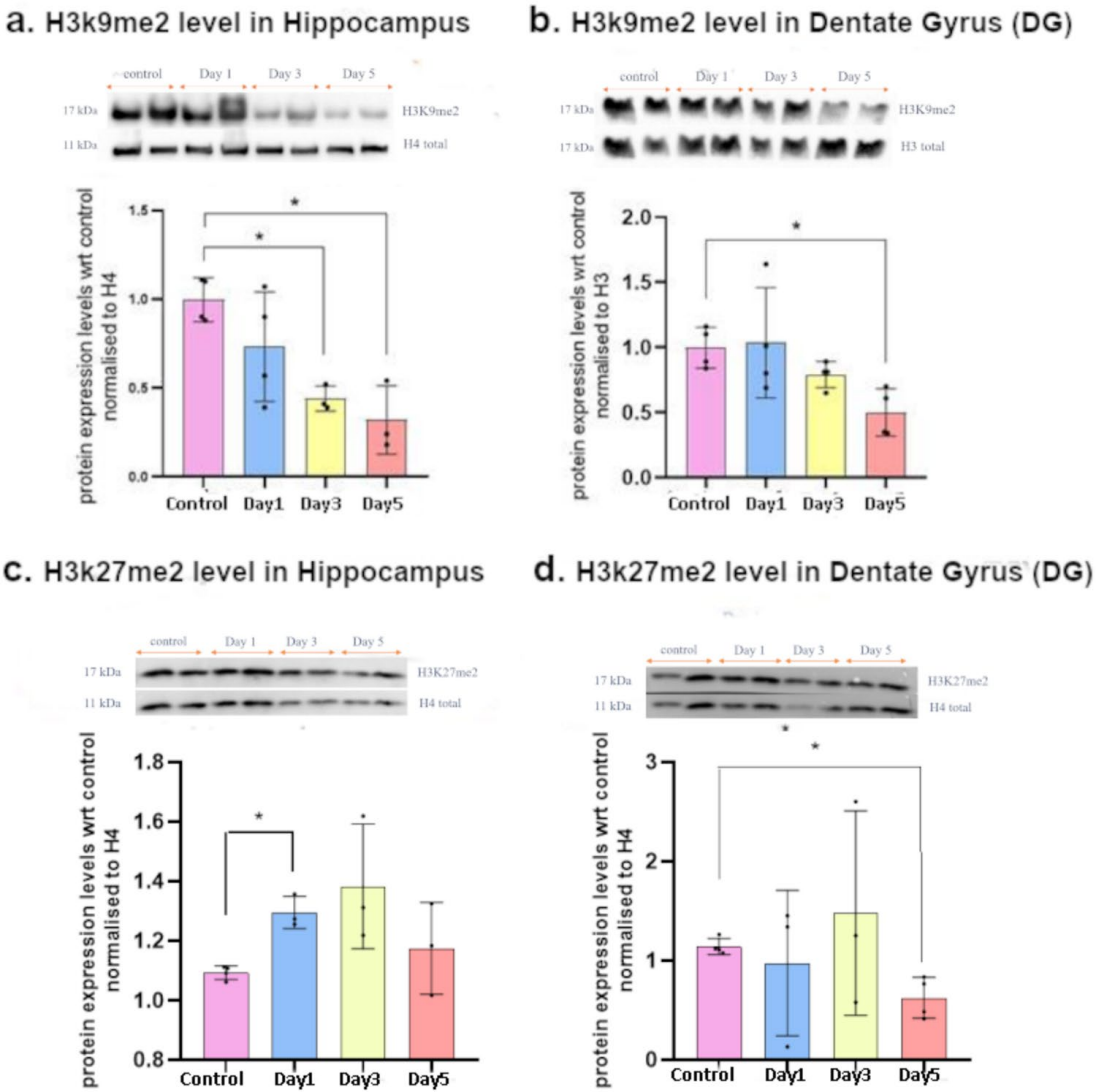
Levels of histone methylation marks measured using western blot after Day 1, Day 3 and Day 5 of the PSDS model: **a** global H3K9me2 levels in the hippocampus; **b** global H3K9me2 levels in the DG; **c** global H3K27me2 levels in the hippocampus; **d** global H3K27me2 levels in the DG; Blots shown are representative and graphs show densitometric quantification from multiple blots; Significance calculated using Student’s *t*-test; *p ≤ 0.05; data presented as mean ± SD (*n* = 3–4)

Dynamic changes in histone methylation marks have been observed in models of stress and depression. Alterations in repressive histone lysine methylation marks have been seen in many brain regions involved in depression, like the nucleus accumbens (NAc), prefrontal cortex (PFC) and also the hippocampus. Notable among these are repressive marks associated with H3K9 and H3K27, which have been reported to be dysregulated in the brain in depression models in many studies, including previous ones from our lab (Covington et al. 2011; Wilkinson et al. 2009; Jiang et al. 2021; Hunter et al. 2009; Pathak et al. 2017). To understand the onset of repressive histone methylation-based epigenetic reprogramming as a result of progressive stress, we used western blot analysis to measure the global levels of H3K9me2 and H3K27me2 in the DG and the rest of the hippocampus. We observed a significant reduction in H3K9me2 levels in the DG after the 5th stress event, and a significant reduction of the same in the rest of the hippocampus after the 3rd stress event (Fig. 3a, b). In the case of H3K27me2 too, we observed a significant reduction in the DG after the 5th stress event, but a significant increase was observed in the rest of the hippocampus after the 1st stress episode followed by an upward trend in expression with stress progression (Fig. 3c, d). The finding suggests that a reduction in repressive H3K9me2 and H3K27me2 methylation in the DG correlates with the advent of the behavioral phenotype of depressive disorder.

**Fig. 4:**
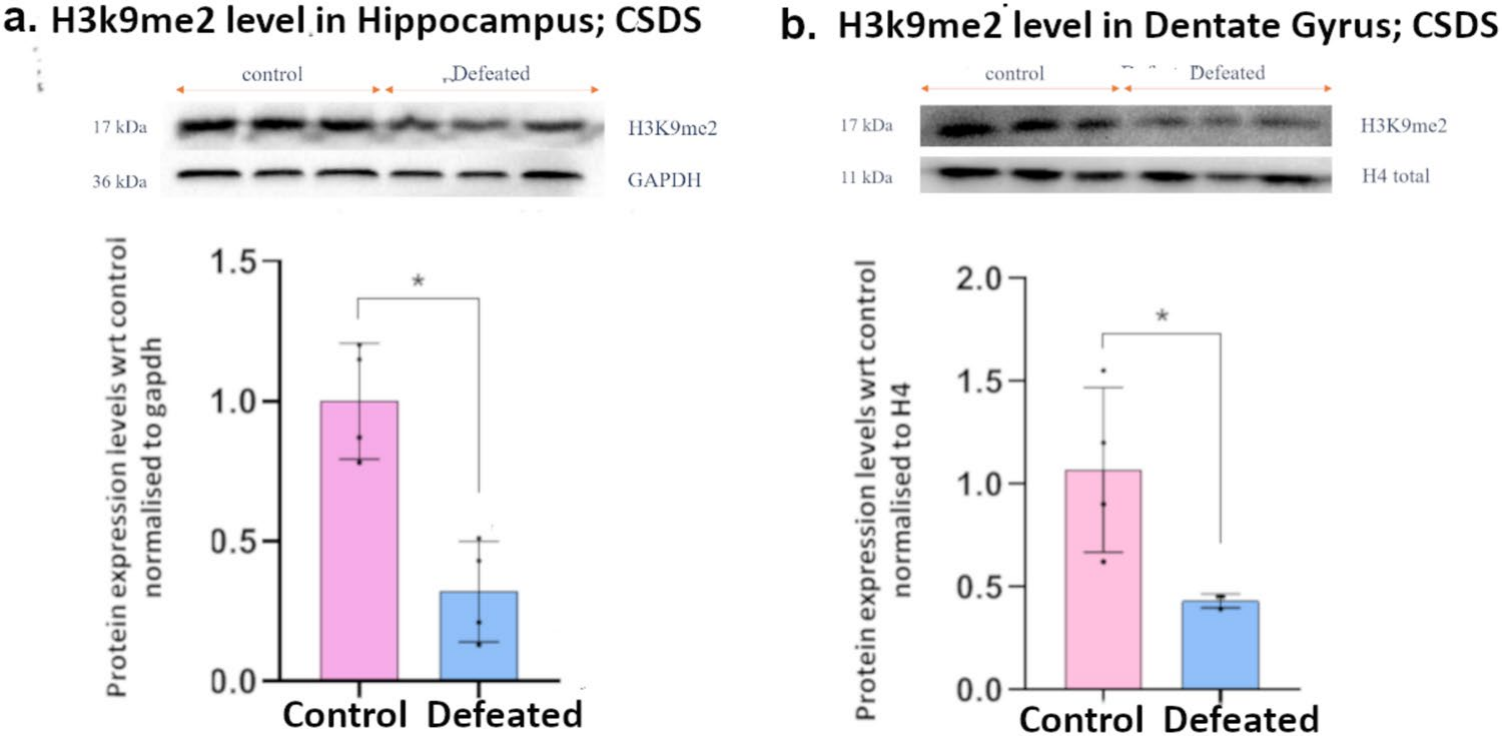
Levels of histone methylation marks measured using western blot after 10 days of CSDS: **a** global H3K9me2 level in the hippocampus; **b** global H3K9me2 level in the DG; Blots shown are representative and graphs show densitometric quantification from multiple blots; significance level was calculated using Student’s *t*-test; *p ≤ 0.05; data presented as mean ± SD (*n* = 3–4)

Furthermore, we found the reduction in H3K9me2 levels to be consistently present even after 10 days of CSDS (Fig. 4a, b), indicating that these epigenetic changes play an important role in establishment and maintenance of depression-like phenotype.

### Altered Transcript Levels of Histone Lysine Methylases and Demethylases in the Hippocampus in the Progressive Social Defeat Stress Model

**Fig. 5:**
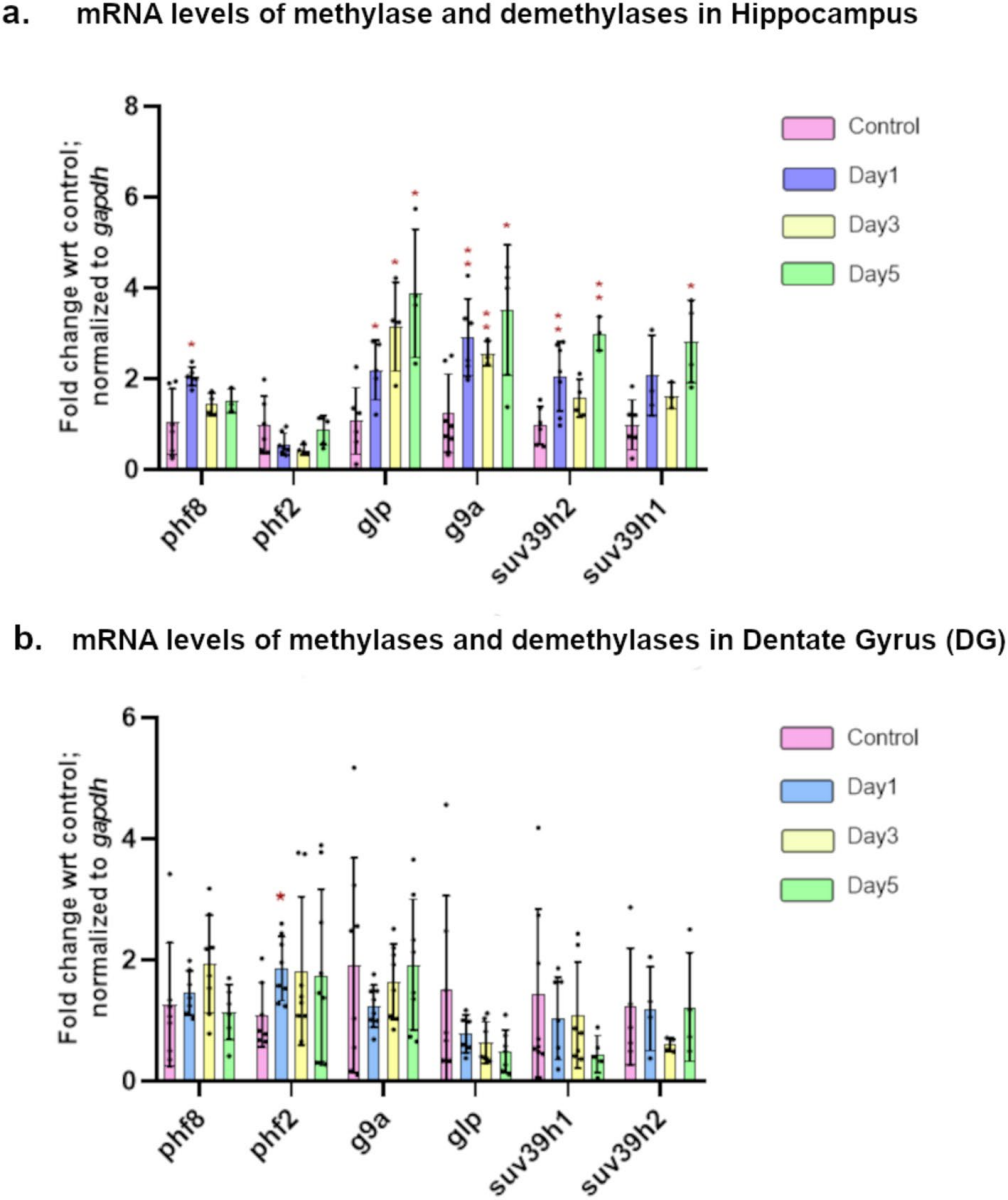
Expression levels of histone modifying enzymes, methylases and demethylases after Day 1, Day 3 and Day 5 of the PSDS model in **a** hippocampus; **b** DG; graphs show relative fold change in mRNA of the genes; *gapdh* was considered as the housekeeping control; significance level was calculated using Student’s *t*-test; *p ≤ 0.05; **p ≤ 0.01; data presented as mean ± SD (*n* = 7–9)

Based on the previous observations, we measured the mRNA expression levels of methylases and demethylases, in both the DG and the rest of the hippocampus, to gain a better understanding of the mechanisms that are involved in the repressive methylation and demethylation of histones. We quantified the expression levels of demethylases such as *phf8, phf2* and methyltransferases such as *glp, g9a, suv39h2* and *suv39h1*, all of which are known to target H3K9me2/me3 marks; using RT-qPCR technique. We observed that in the hippocampus, *phf8* showed a significant upregulation after the first stress event, and then its expression dropped to the same level as that of control, although *phf2* did not show any significant changes throughout the stress paradigm. Both *glp and g9a* showed a consistent and significant increase in expression with stress progression, starting from Day 1 of stress and a similar pattern was observed for *suv39h1*. *suv39h2* showed a significant increase after the 3rd day of stress (Fig. 5a). Despite the increase in the expression of methyltransferases *glp*, *g9a*, *suv39h1* and *suv39h2*, which are known to deposit H3K9me2 (Mozzetta et al. 2014), the levels of H3K9me2 and H3K27me2 marks were found to be decreased after the 4th and 5th stress episodes. This could be because of the transient increase in expression seen in *phf8* that demethylates H3K9me2 (Qi et al. 2010) is seen after the first stress episode.

When we quantified the expression of these genes in the DG, a significant upregulation of *phf2* was observed after Day1 of stress that maintained an upward trend up to Day 5 of stress; a clear downward trend was observed in the expression of *glp*, starting after the first stress episode. While *phf8* showed an upward expression trend up to the 3rd stress event, *suv39h1* showed a downward expression trend after 4th and 5thstress events (Fig. 5b). The transient increase in *phf2* expression along with the increasing trend in the expression of *phf8* and the decreasing trend in *glp* can explain the reduced H3K9me2 levels in the DG after 4 and 5 stress episodes.

All these results also suggest that changes in histone modifying enzymes pre-empts the changes seen in global histone methylation levels and the depression-like phenotype.

## Discussion

Chronic stress can lead to depression by affecting molecular pathways in the brain (Tafet and Bernardini 2003; McGonagle and Kessler 1990). This has formed the basis for the development of animal models of stress-induced depression, and the chronic social defeat stress (CSDS) model is one of the most popular ones used to study depression (Golden et al. 2011). Epigenetic factors are intricately involved in mediating stress effects on the brain (Mifsud et al. 2011), with a lot of evidence of altered epigenetic marks and factors as a consequence of chronic as well as acute stress (Uddin et al. 2010; Bilang‐Bleuel et al. 2005). In order to understand the progression of stress effects on depression related behavioral phenotype and the underlying epigenetic modifications in the brain, we developed a modification of the CSDS model, the progressive social defeat stress (PSDS). In this model, we observed that the classical signs of depression-like behavior start manifesting after four defeat stress events. In a previous study from our lab, we have already shown that five days of defeat stress is sufficient to induce depression-like phenotype in mice (Pathak et al. 2017). A more recent study has also reported establishment of depression-like phenotype after four days of defeat stress, evidenced by an increase in immobility period in forced swimming test (Harada et al. 2023).

Further, based on compelling evidence implicating the hippocampus and hippocampal neurogenesis (Fuchs et al. 2004) in depression, we decided to specifically look at this brain region. In the PSDS model, we found that a marker of early neuronal differentiation, *dcx* was significantly downregulated in the neurogenic niche of the hippocampus, the dentate gyrus (DG) after five events of defeat stress. Additionally, an increasing trend in its expression levels was observed up to three stress events. Such dynamic changes in neurogenesis in response to stress have been previously reported. Lagace et al. (2010) have shown that only a transient decrease in hippocampal neurogenesis occurs after chronic stress, with mice showing persistent social avoidance having higher surviving neurons in the DG. Other studies, including one from our lab have shown an attenuation of hippocampal neurogenesis after 10 days of CSDS (Surget et al. 2008; Sun et al. 2018; Maitra et al. 2020). Here, we report attenuation after 5 days of defeat stress. This is in line with a previous finding where a decrease in *dcx* positive neurons in the ventral hippocampus was noted (Harada et al. 2023). This suggests that stress related molecular changes that cause an advent of depression-like phenotype might also be involved in altering hippocampal neurogenesis.

Repressive histone methylation modifications, H3K9me2 and H3K27me2 have been extensively reported to be altered in brain regions associated with stress and depression and implicated in neurogenesis as well (Maitra et al. 2020; Guerra et al. 2022; Harutyunyan et al. 2019). In the PSDS model, we observed a significant decrease in global levels of H3K9me2 after the fifth and third stress events in the DG and the rest of the hippocampus, respectively. On the other hand, H3K27me2 showed a significant decrease after the fifth day of stress in DG, while in the hippocampus, it showed a significant increase after the first day of stress and maintained an upward trend till the fifth day of stress. We further noted that the reduced levels of H3K9me2 in the DG and the hippocampus remain even after 10 days of defeat stress. A previous study has shown an increase in H3K9me2 levels in the hippocampus of maternally separated rats that underwent unpredictable mild stress in adulthood (Jiang et al. 2021). For the first time, we have shown a persistent decrease in H3K9me2 levels in the DG and the rest of the hippocampus as a result of defeat, suggesting its involvement in the establishment and maintenance of depression-like phenotype.

We further measured the expression levels of a few histone modifying enzymes involved in methylating and demethylating H3K9me2. We observed an upregulation of phf8 in the hippocampus after the first day of stress and a trend of the increase was maintained till the third day of stress. We also observed an upregulation of phf2 in the DG after the first day of stress and this increasing trend is maintained till the fifth day of stress, which might be responsible for the decreased level of H3K9me2 levels in DG and hippocampus after the third day of stress. Methyltransferases, such as *glp*, *g9a* and *suv39h2* showed an increased level of expression in hippocampus, after first day of stress and suv39h1 showed an increased level expression after the third day of stress; we predict that the increased levels of phf8 during the early stress event, is responsible for the reduced levels of H3K9me2 marks. In DG, we observed a downward trend in expression levels of *glp* and *suv39h2* which corresponds with the H3K9Me2 and H3K27me2 levels in DG. Further in-depth study is required to understand the histone methylation and demethylation patterns across the genome and their relation with levels of different methylases and demethylases.

Similar to hippocampus and DG, nucleus accumbens (NAc) which is the reward center of the brain has also been heavily implicated in MDD (Misaki et al. 2016; Pizzagalli et al. 2009). In a previous study from our lab we observed that after 5 days of CSDS paradigm, *jmjd2a* family of histone demethylase and H3K36me2 histone marks show differential expression among, control, resilient and susceptible animals with a marked increase in their abundance in susceptible animals while their levels remain comparable to the controls in case of the resilient group (Pathak et al. 2017). Also previously unpublished data from our lab shows a transient differential expression of *phf2* between the susceptible and resilient groups of animals, with a significant upregulation in resilient group where the susceptible group shows a downregulation, this change is lost by the 10th day of the CSDS protocol where the expression of *phf2* in the susceptible and resilient group become comparable to that of control (Sup. Figure 1a, b). Similarly, the methyltransferases, *g9a* and *suv39h1* show transient upregulation and downregulation respectively after 5 days of stress paradigm, while after 10 days of stress paradigm we had observed a different expression pattern where the *g9a* expression level becomes similar in all three groups and the susceptible group shows a marked upregulation in *suv39h1’s* expression (Sup. Figure 2a, b).

From these findings, it can be concluded that epigenetic changes occur earlier than the behavioral phenotype of depression. And many such molecular changes that occur in brain during early stress are transient in nature and are relative to the number of stress events that has been undergone by the animal, also from our study it can be predicted that dynamic changes in histone methylation accumulates across different regions of the brain during stress, which leads to depression-like phenotype over a period of continuous stress; also these transient changes and the individual variations of these histone methylase and demethylase genes among the animals might be a determinant factor for Stress susceptibility or resilience. Overall, the PSDS protocol will be a useful tool to understand the molecular background of stress progression into depression-like phenotype, and studying the transient molecular changes might prove useful as an early stage marker of stress progression, allowing better and more rapid intervention.

## Methodology

All animal experiments were performed as per the approval of the institutional animal ethics committee of CSIR-CCMB, IEAC 20/GO/RBi/99/CPCSEA.

### Chronic Social Defeat Stress Protocol

The 10 days chronic social defeat stress (CSDS) paradigm is a well-established paradigm for inducing depression-like phenotype in mice; which is comparable to humans (Krishnan et al. 2007; Berton et al. 2006). We used 6- to 8-week-old C57/BL6 Ncrl (henceforth mentioned as C57) mice as experimental animals for this study; the animals were housed and maintained in the animal facility of Center for Cellular and Molecular Biology. CD1 retired breeders of 7–8 months age were used as aggressors for the protocol. Aggressors were generated by isolating them by housing them in one half of rat cages separated using perforated plexiglass separators into two halves. They were tested for aggressive behavior using C57 mice that were excluded from the experiment. During the experiment, the C57 experimental mice were housed individually on the opposite side of the separator from the aggressor for the mice to be subjected to stress and control animals were housed individually with C57 control mice on either side of the separator. Every 24 h during the experimental procedure, the C57 mice were introduced to the territory of the aggressor mice for 5 min, during this time this “intruder” mouse was subjected to attacks and antagonistic behavior from the aggressor. After the 5 min of interaction the experimental mice was physically separated from the aggressor, on the other side of the separator. The animals still maintained visual and olfactory contact, causing the experimental animals to still feel threatened. This process was repeated for 10 days, during which each day the experimental animal was exposed to an unfamiliar CD1 aggressor, to avoid familiarity. The non-stressed C57 control mice were allowed to interact with each other for 5 min every 24 h instead of the aggressor for the duration of the paradigm.

### Progressive Social Defeat Stress Paradigm

This is a variant of the classic social defeat stress (SDS) paradigm that was devised by our lab to study stress progression into depression-like phenotype. To this end, we generated different groups of C57 mice that were subjected to SDS for different number of days up to a maximum of 5 days, as previous data from our lab suggests 5 days of SDS being sufficient for developing depression-like phenotype in C57 mice (Pathak et al. 2017).

**Fig. 6:**
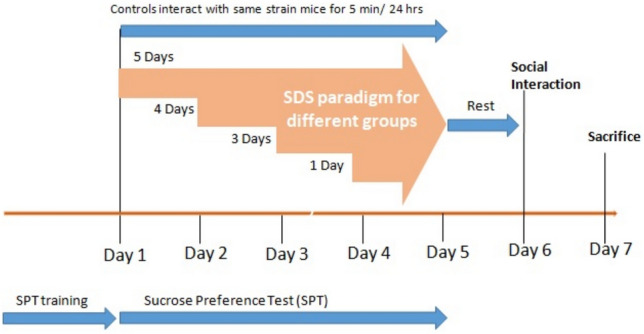
Progressive social defeat stress protocol; each group of animals is named after the total number of days they undergo the SDS paradigm; i.e., 1 Day, 2 Days, 3 Days, 4 Days and 5 Days, respectively; during the gap between the final stress event and sacrifice, animals were housed in the same cages as their aggressors on the other side of the separator

We generated 6 groups of C57 mice: control group, which was not subjected to defeat stress, and 5 experimental groups that had been subjected to social defeat stress by CD1 aggressors for a variable number of days in a progressive manner, from 1 day through 5 days of stress. The animals in the control non-stressed group were allowed only regular interaction with other C57 mice during the full duration of the experiment. social interaction test was performed on the 6th day and the animals were sacrificed on the 7th day (Fig. 6).

### Social Interaction Test

The social interaction test was performed to assess the social avoidance behavior for unfamiliar target (aggressor). The mice were individually introduced to an open arena of dimensions 47 × 37 cm^2^; with a social interaction box; a clear perforated plexiglass box of dimensions 10 × 10 cm^2^ (Fig. 2a) which was placed in the middle of one of the shorter edges of the arena. Using the *EthoVision* 3.1 software the arena was virtually divided into 3 separate zones; an interaction zone marked around the plexiglass interaction box, while the two corners of the arena opposite of the interaction zone was marked as corner zone.

The C57 experimental mice were released individually into the arena for 5 min each, first time while the interaction box was empty (without target) and second time while the interaction box housed an unfamiliar CD1 aggressor (with target). The movements of the experimental animal within the arena were tracked for the 5 min duration of both the trials for each animal, using EthoVision.

The Interaction percentage was calculated as follows

$$\left[ {\left\{ \begin{gathered} {\text{Time spent in the interaction zone in presence of target }}\left( {{\text{2nd trial}}} \right)/ \hfill \\ {\text{total time spent in the interaction zone in presence and absence of target }}\left( {{\text{1st trial}} + {\text{2nd trial}}} \right) \hfill \\ \end{gathered} \right\}~ \times ~{\text{1}}00} \right]$$

### Sucrose Preference Test

Anhedonia, which is the decrease in the ability to elicit pleasure from otherwise pleasurable activities is a classic symptom of depression in humans (Tuohy and McVey 2008; Vahia 2013). In mice it has been reported that mice subjected to chronic stress show signs of anhedonia. Normally mice prefer to consume water containing 2% sucrose compared to plain drinking water. However stressed mice showing depression-like phenotype show a marked change in their preference for 2% sucrose solution, showing a reduced consumption of the same (Katz 1982).

For this test 4 days before the stress paradigm all mice were provided with plain drinking water in two specially made 10ml sipper pipettes. Next day both the pipettes were switched for 2% sucrose solution. After which from the following day the mice were given a choice between 2 pipettes, one containing plain drinking water and other containing 2% sucrose solution, the position of the two pipettes were interchanged each day and their contents were refilled, after taking measurements to determine the amount of each liquid consumed by the mice. This process was repeated for the duration of the stress paradigm to observe the progression of stress.

Sucrose preference of the mice was calculated using the following formula

$$\left[ {\left\{ \begin{gathered} {\text{Volume}}\;{\text{of}}\;{\text{ 2}}\% \;{\text{sucrose}}\;{\text{solution}}\;{\text{consumed}}/{\text{total}}\;{\text{volume}}\;{\text{of}}\;{\text{liquid}}\;{\text{consumed}} \hfill \\ \;\;\;\;\left( {{\text{volume}}\;{\text{of}}\;{\text{sucrose}}\;{\text{consumed}}\; + \;{\text{volume}}\;{\text{of}}\;{\text{water}}\;{\text{consumed}}} \right) \hfill \\ \end{gathered} \right\}\; \times \;{\text{1}}00} \right]$$

#### Sacrifice of Experimental Animals and Collection of Separate Brain Regions

24 h after the last behavioral test the animals were sacrificed via cervical dislocation. The mice were decapitated and the brain was removed immediately and placed in ice cold autoclaved 1× PBS. The brain was then sliced into 1 mm thick slices, using brain matrix (Zivic rodent brain slicer matrix). Using 12 and 16 gauge needles punches of dentate gyrus (DG) and the rest of the hippocampus were taken separately. The brain sections were collected in 1.5 ml tubes and immediately snap frozen in liquid nitrogen and stored at – 80 °C.

#### RNA Isolation and Gene Expression Studies

RNA was isolated using RNAisoPlus (Takara), according to manufacturer’s instruction. Equal amounts of RNA samples were then used for cDNA synthesis using Primescript 1st strand Synthesis kit (Takara). Quantitative Real-Time PCR was performed in triplicates using TB-Green II (Takara). The mRNA levels were normalized using the mRNA expression levels of the housekeeping gene *gapdh* relative expression of the target genes were calculated using the − ΔΔ*C*_t_ method.

#### Protein Isolation and Immunoblotting

Protein was isolated from the organic phase formed during RNA isolation using RNAiso Plus (Takara). By first removing any DNA contamination with 100% ethanol treatment, followed by precipitation of the protein using Acetone. The pellet was dissolved in urea buffer and 20 μg of the protein was electrophoresed on a 12% poly acrylamide SDS gel. The protein was then transferred onto a PVDF membrane followed by blocking with 5% BSA, and were incubated with appropriate antibodies (Table 1).

**Table 1:** List of antibodies used for immunoblotting, detection and analysis

Name	Produced by	Dilution
H3K9me2 (mouse monoclonal) (cat: ab1220)	Abcam	1:1000
H3K27me2 (rabbit polyclonal) (cat: ab24684)	Abcam	1:1000
H4 pan (rabbit polyclonal) (cat: ab10158)	Abcam	1:1000
Goat anti mouse IgG (HRP) (cat: ab97265)	Abcam	1:3000
Goat anti rabbit IgG (HRP) (cat: ab97200)	Abcam	1:3000

The HRP was then visualized through chemiluminescence using Clarity Max ECL Western Blotting substrate (Biorad), and the intensity was measured using the ImageJ software; to determine the abundance of different methylated histone families.

### Immunohistochemistry

Both groups of animals were intracardially perfused with 4% paraformaldehyde 24 h after the final day of the PSDS. The brains were dissected out and preserved in 30% glycerol solution. 30 µm coronal sections were taken and hippocampal sections and were serially collected in 6 wells in PBS; each well contained every 7th section. From each well, sections were randomly selected. The sections were washed in Milli-Q water and were boiled in citrate buffer (10 mM citrate; 0.05% Tween 20; pH 6.0), for 10 min for antigen retrieval. This was followed by treatment with 0.1 M NaBH_4_ for 5 min and 0.3% Triton X-100 for 45 min. The slices were then incubated with 4% serum for 2 h for blocking. The slices were then incubated in Doublecortin polyclonal rabbit antibody (Abcam ab18723) at 1:1000 dilution prepared in 2% serum overnight. The sections were then washed in PBS (0.1% Triton X-100) three times for 5 min.; after which the slices were incubated in Alexa555 conjugated anti-rabbit secondary antibody (Abcam ab150090) prepared in 2% serum, followed by PBS (0.1% Triton X-100) wash. The sections were then incubated in 1% DAPI for 5 min and washed in PBS (0.1% Triton X 100) without shaking. The slices were then mounted on positively charged slides and observed under Leica SP8 confocal microscope under ×20 oil immersion lens, and the images were analyzed using ImageJ.

Fluorescence intensity was calculated by standard corrected total cell fluorescence (CTCF) measurement. The images were converted to 8-bit followed by marking the area to be measured—the subgranular zone of the dentate gyrus. For each image, background fluorescence was calculated too. The parameters, mean, minimum and maximum gray value and integrated density were measured. CTCF was calculated using the following formula:

$${\text{CTCF}}\; = \;{\text{integrated}}\;{\text{density}}\;{-}\;\left( \begin{gathered} {\text{area}}\;{\text{of}}\;{\text{selected}}\;{\text{cell}} \hfill \\ \times \;{\text{nean}}\;{\text{fluorescence}}\;{\text{of}}\;{\text{background}}\;{\text{readings}} \hfill \\ \end{gathered} \right)$$

### Statistical Test

To analyze statistical significance between different groups, we performed student’s *t*-test to compare each stress group to the control individually to understand the difference between the control animals and animals subjected to progressive stress paradigm. A *p*-value below 0.05 but above 0.01 is represented by a single asterisk above the graph, while a *p*-value below 0.01 is represented by two asterisks above the graph.

## Competing interests

The authors declare no competing interests.

## Supplementary Information

Below is the link to the electronic supplementary material.

Supplementary file1 (DOCX 326 kb)

## Data Availability

No datasets were generated or analysed during the current study.
