# Supplementary material for: Role of Repressive Histone Lysine Demethylases and Methylases in Susceptibility to Depression Using a Novel Progressive Social Defeat Stress Mouse Model
Source: Cell Mol Neurobiol. 2025 Aug 11;45:78. doi: 10.1007/s10571-025-01597-3 (PMC12339800; doi:10.1007/s10571-025-01597-3)
Supplement: Supplementary file 1 — Supplementary file1 (DOCX 326 kb) [file 10571_2025_1597_MOESM1_ESM.docx]

**Role of repressive histone lysine demethylases and methylases in susceptibility to depression using a novel progressive social defeat stress mouse model**

Arpan Mukhoti^#1,3^, Annapoorna PK^#1,3^, Ashutosh Kumar^1^, Pratishtha Wadnerkar^1^, Ayesha Atqa Khan^1^, Salil Saurav Pathak^1^, Sumana Chakrvarty^2,3^, Arvind Kumar*^1,3^

^1^CSIR-Centre for Cellular and Molecular Biology, Hyderabad

^2^Applied Biology Division, CSIR- Indian Institute of Chemical Technology, Hyderabad

^3^Academy of Scientific and Innovative Research, Ghaziabad, UP-201002`

#These authors contributed equally

**Running title:** Repressive methylation and stress susceptibility in PSDS

*Corresponding author:

Dr. Arvind Kumar

Epigenetics & Neuropsychiatric Disorders Laboratory

CSIR-Centre for Cellular and Molecular Biology

Uppal Road, Habsiguda, Hyderabad 500007, Telangana State

Email: [akumar@ccmb.res.in](mailto:akumar@ccmb.res.in); [arvindk567@gmail.com](mailto:arvindk567@gmail.com)

Phone: +91 40 27192826

**Supplementary Figures:**


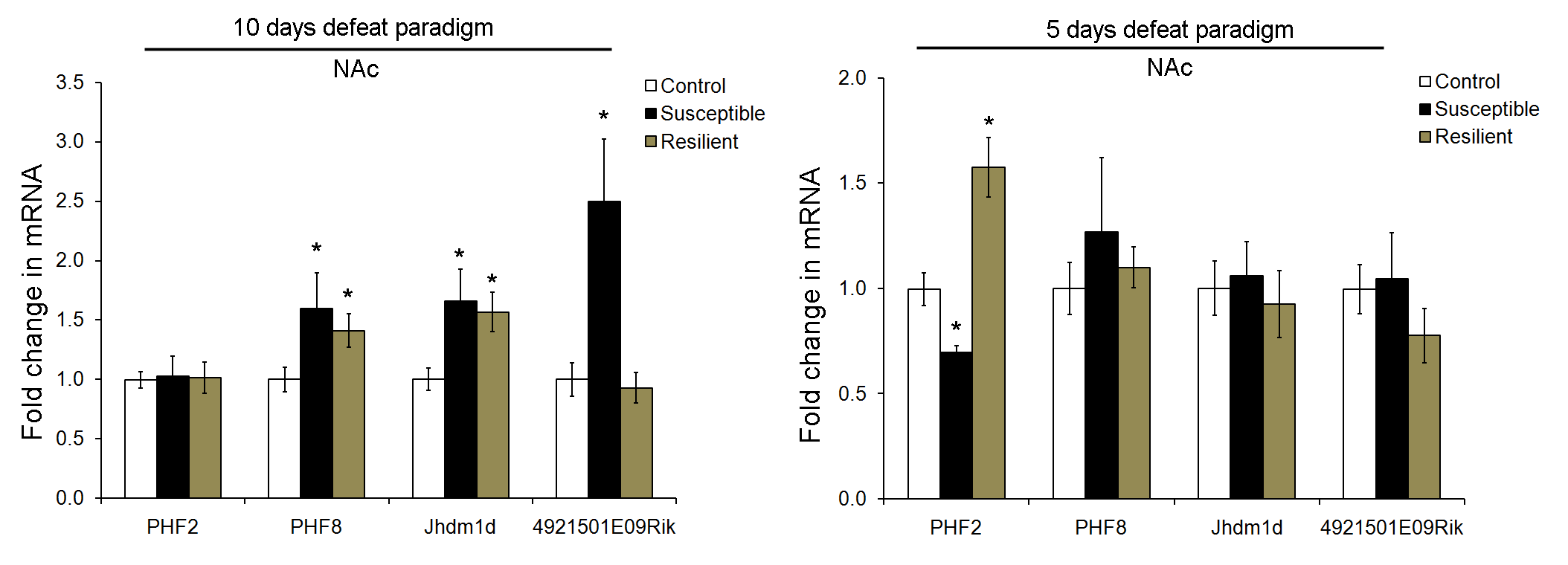

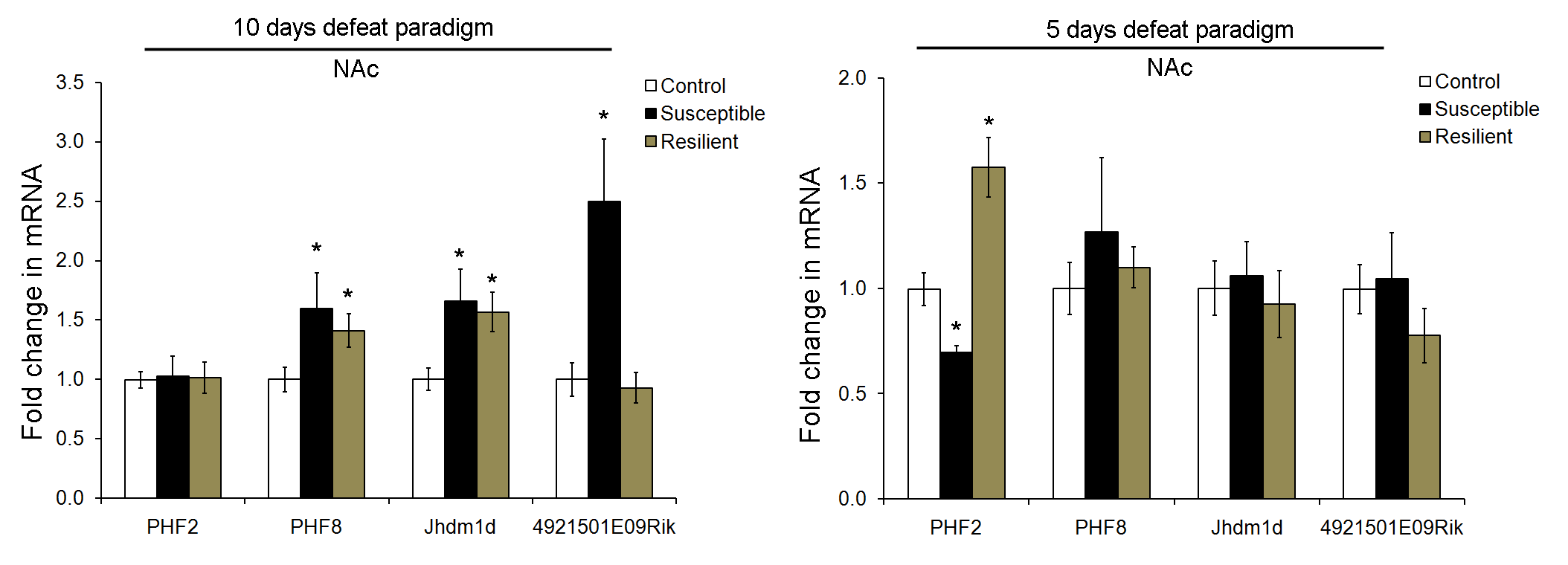


a.

b.


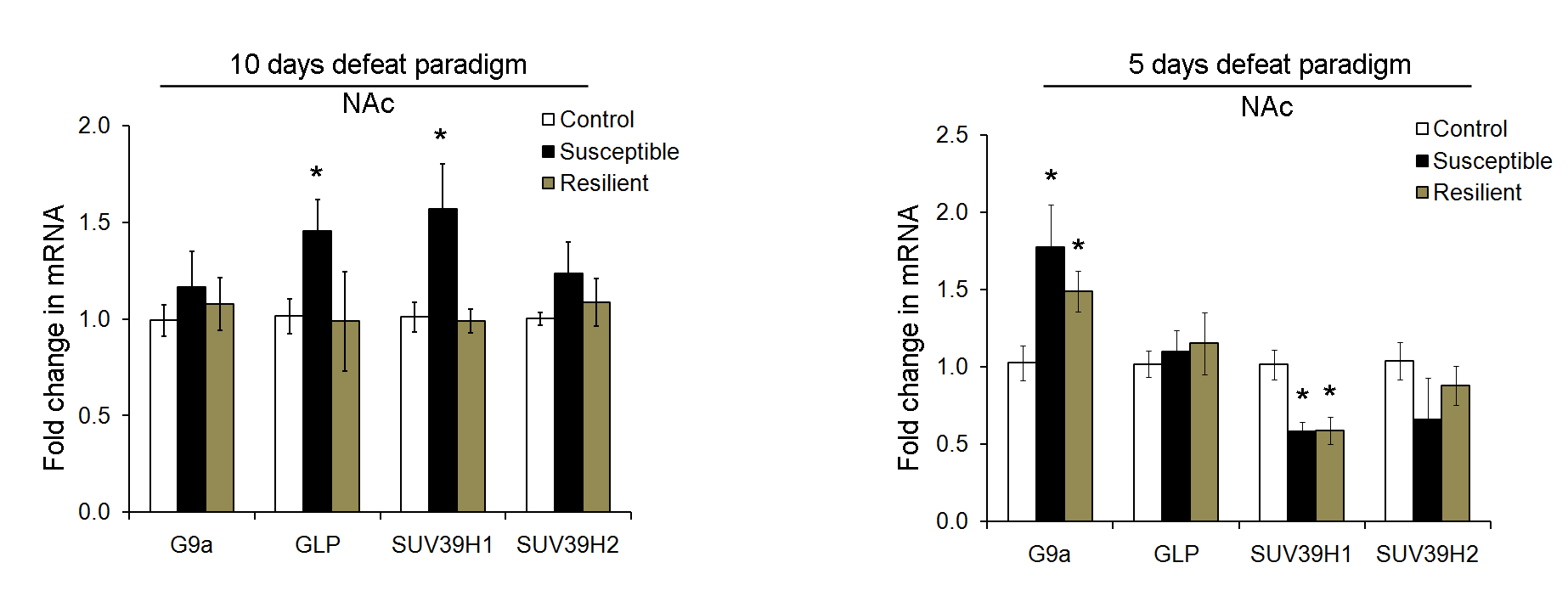

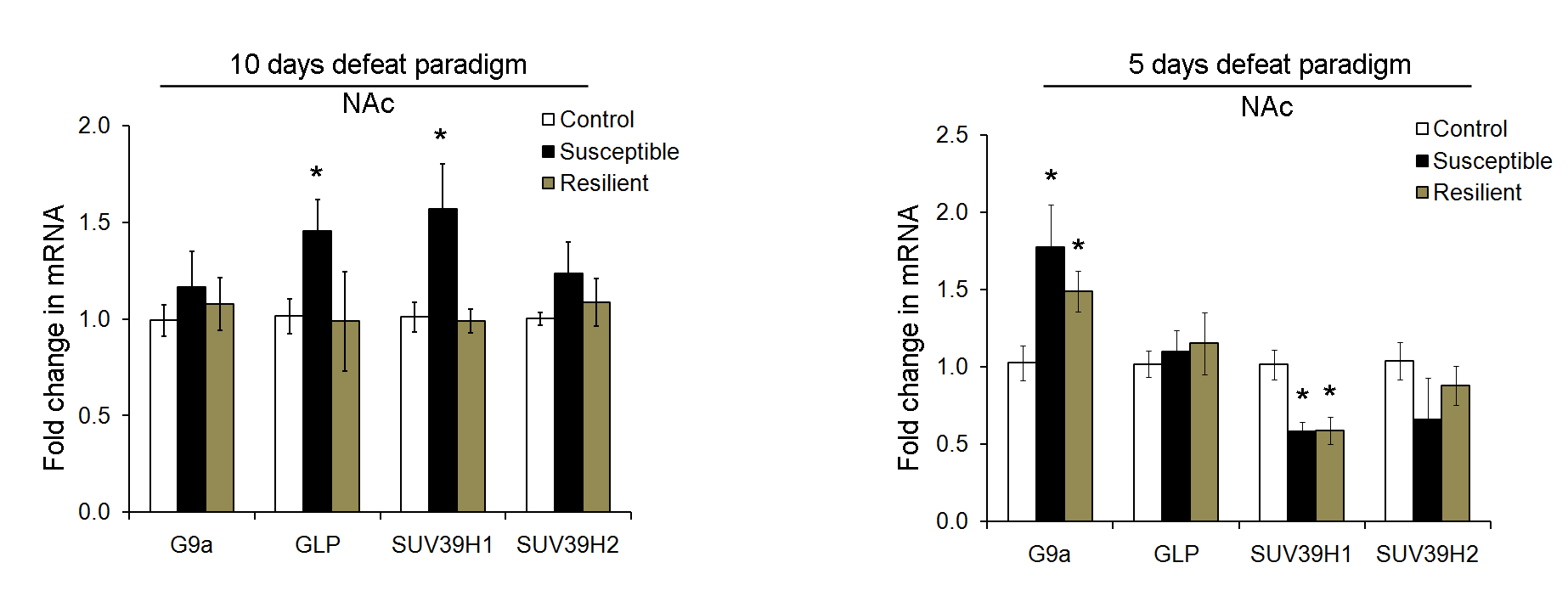


**Supp Fig 1:** q-PCR analysis of Histone demethylase expression levels in Nucleus Accumbens(NaC) after (a.) 5 days of CSDS and (b.) 10 days of CSDS; Fold change was calculated using ΔΔcT method, bars represent mean±SE with n=6-12; significance was calculated using student’s TTest; *≤0.05.


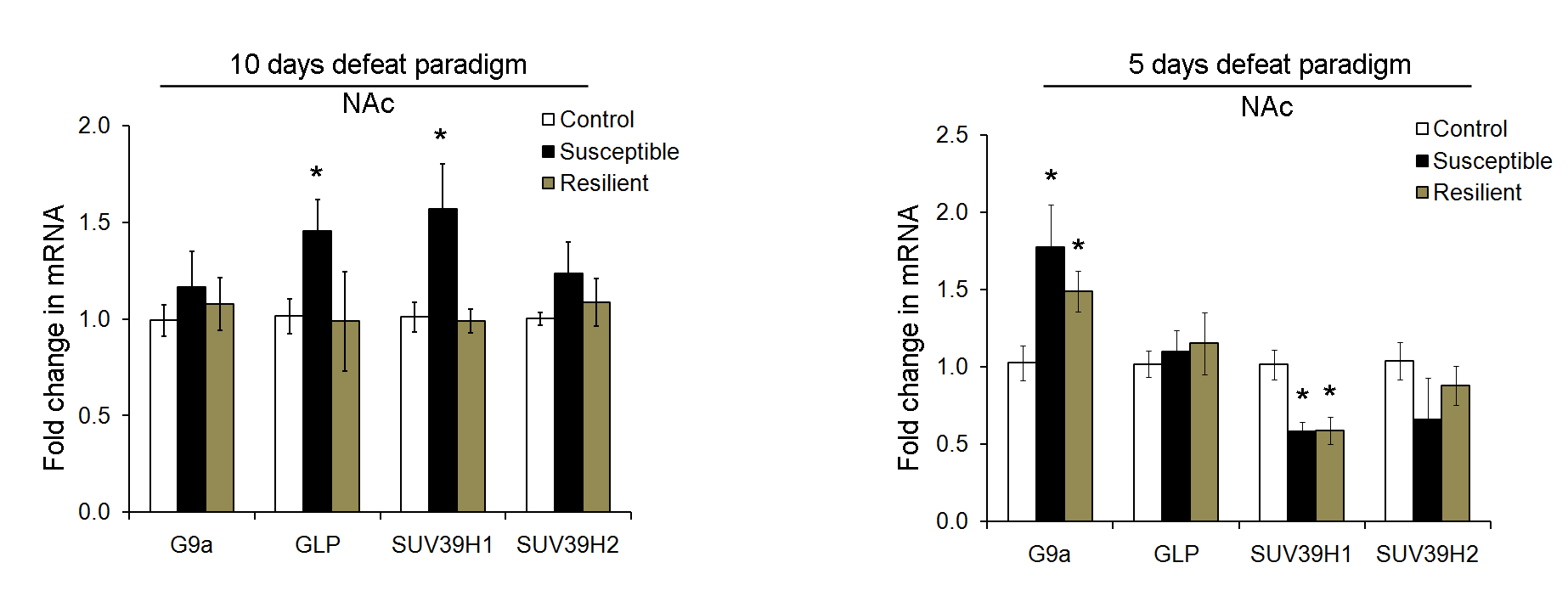

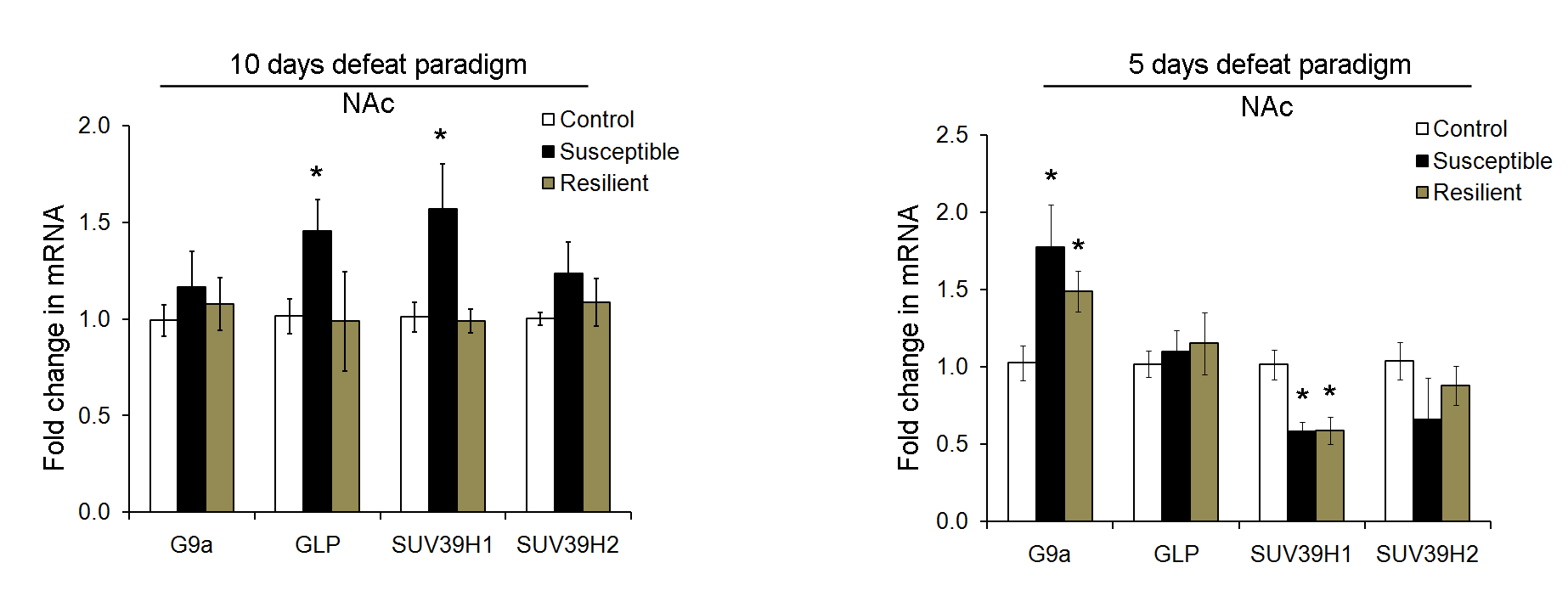


a.

b.

**Supp Fig 2:** q-PCR analysis of Histone methyltransferase expression levels in Nucleus Accumbens(NaC) after (a.) 5 days of CSDS and (b.) 10 days of CSDS; Fold change was calculated using ΔΔcT method, bars represent mean±SE with n=6-12; significance was calculated using student’s TTest; *≤0.05.
